# Characterization of the complete chloroplast genome of *Sinosenecio oldhamianus* (Compositae)

**DOI:** 10.1080/23802359.2019.1675483

**Published:** 2019-10-11

**Authors:** Jingjing Xu, Yihua Gong, Guoqian Hao

**Affiliations:** a Key Laboratory for Bio-resource and Eco-environment of Ministry of Education, College of Life Sciences, Sichuan University, Chengdu, Sichuan, P. R. China;; b Administration Office of Meigu Dafengding National Nature Reserve, Meigu, Sichuan, P. R. China;; c Biodiversity Institute of Mount Emei, Mount Emei Scenic Area Management Committee, Leshan, Sichuan, P. R. China

**Keywords:** *Sinosenecio oldhamianus*, Compositae, chloroplast genome, phylogenetic tree

## Abstract

The complete chloroplast (cp) genome sequence of *Sinosenecio oldhamianus*, a common medicinal plant is widely distributed in South China. The plastome is 150,926 bp in length, with one large single-copy region of 94,588 bp, one small single-copy region of 18,130 bp, and two inverted repeat (IR) regions of 24,852 bp. It contains 134 genes, including 87 protein-coding genes, 8 ribosomal RNA, and 37 transfer RNA. The phylogenetic tree shows that this species is a sister to the genus *Ligularia*. The published plastome within *Sinosenecio* provides significant insight for elucidating the phylogenetic relationship of taxa within tribe Compositae.

*Sinosenecio oldhamianus* (Maxim.) B. Nord., a common medicinal plant (Wu and Wu 2003; Liang and Ye 2006; Yang et al. 2014), belongs to the genus *Sinosenecio* (Compositae), consisting of ∼40 species (Chen et al. 2011). and is widely distributed in South China. Because of the complexity of their inter- and intraspecific morphological variations, species delimitation is sometimes quite difficult. However, no information is available about its molecular biology and no genomic study has been performed on *S. oldhamianus*. In our study, we characterized the complete chloroplast (cp) genome sequence of *S. oldhamianus* for further physiological, molecular, and phylogenetical study of this medicinal species.

Fresh leaves of *S. oldhamianus* were collected from Xiaozhaizigou National Reserve (Mianyang, Sichuan, China; coordinate: 103°45′E, 31°50′N), dried and kept in silica gel for DNA extraction, and then stored in the College of Life Science, Sichuan University. Total genomic DNA was extracted with a modified CTAB method (Doyle and Doyle 1987). First, we obtained 10 million high-quality pair-end reads for *S. oldhamianus*, and after removing the adapters, the remaining reads were used to assemble the complete cp genome by NOVOPlasty (Dierckxsens et al. 2017). The complete cp genome sequence of *Sinosenecio polylepis* was used as a reference. Plann v1.1 (Huang and Cronk 2015) and Geneious v11.0.3 (Kearse et al. 2012) were used to annotate the cp genome and correct the annotation.

The *S. oldhamianus* cp genome is 150,926 bp in length, exhibits a typical quadripartite structural organization, consisting of a large single-copy (LSC) region of 83,092 bp, two inverted repeat (IR) regions of 24,852 bp each, and a small single-copy (SSC) region of 18,130 bp. The cp genome contains 134 complete genes, including 87 protein-coding genes (87 PCGs), 8 ribosomal RNA genes (4rRNAs), and 37 tRNA genes (37 tRNAs). Most genes occur in a single copy, while 17 genes occur in double, including 4 rRNAs (*4.5S*, *5S*, *16S*, and *23S rRNA*), 7 tRNAs (*trnA-UGC*, *trnI-CAU*, *trnI-GAU*, *trnL-CAA*, *trnN-GUU*, *trnR-ACG*, and *trnV-GAC*), and 6 PCGs (*rps7*, *rpl2*, *rpl23*, *ndhB*, *ycf2*, and *ycf15*), while a partial *ycf1* and *rps19* genes were identified at the IRb/SSC junction as a pseudogene. The overall AT content of cp DNA is 62.7%, while the corresponding values of the LSC, SSC, and IR regions are 64.6, 69.4, and 57.0%.

In order to further clarify the phylogenetic position of *S. oldhamianus*, the plastomes of five representative Compositae species were obtained from NCBI to construct the plastome phylogeny, with *Sonchus boulosii* as an outgroup. All the sequences were aligned using MAFFT v.7.313 (Katoh and Standley 2013) and maximum-likelihood phylogenetic analyses were conducted using RAxML v.8.2.11 (Stamatakis 2014). The phylogenetic tree shows that *S. oldhamianus* clustered together with *Ligularia hodgsonii*, the *Artemisia selengensis* clustered together with *Opisthopappus taihangensis* and *Chrysanthemum lucidum*, forming one clade within the Compositae (Figure 1).

**Figure 1.**
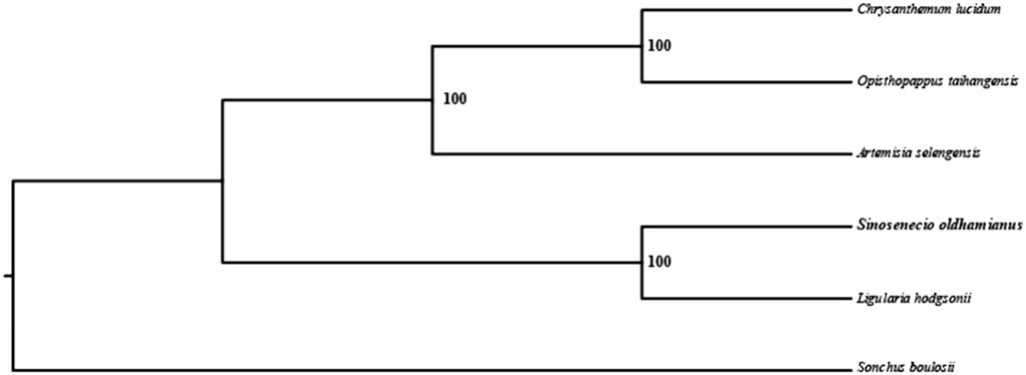
Phylogenetic relationships of Compositae species using whole chloroplast genome. GenBank accession numbers: *Artemisia selengensis* (NC_039647), *Chrysanthemum lucidum* (NC_040920), *Ligularia hodgsonii* (NC_039381), *Opisthopappus taihangensis* (*MK552323*), *Sonchus boulosii* (NC_042244).
